# Understanding the Impact of Extrusion Treatment on Cereals: Insights from Alterations in Starch Physicochemical Properties and In Vitro Digestion Kinetics

**DOI:** 10.3390/ani14213144

**Published:** 2024-11-02

**Authors:** Yufei Zhao, Xiuquan Dang, Honglin Du, Dapeng Wang, Jiaxuan Zhang, Rujie Liu, Zhenying Ge, Zewei Sun, Qingzhen Zhong

**Affiliations:** Jilin Province Key Laboratory of Animal Nutrition and Feed Science, College of Animal Science and Technology, Jilin Agricultural University, Changchun 130118, China; zhao823207830@163.com (Y.Z.); 13353216123@163.com (X.D.); 18844013288@163.com (H.D.); dpwang1017@hotmail.com (D.W.); jasonchang0817@outlook.com (J.Z.); rujieliu@126.com (R.L.); zhenyingge@yeah.net (Z.G.)

**Keywords:** cereal, digestive dynamics, extrusion, physicochemical properties

## Abstract

In this study, three samples were randomly selected from corn, wheat, and broken rice before and after extrusion for electron microscope scanning, Fourier transform infrared spectral analysis, and in vitro digestion to investigate the impact of extrusion on physicochemical characteristics and starch digestion kinetics of cereals. The cereals used for extrusion were sourced identically before and after the process, with each analysis conducted in triplicate. The results showed that the extrusion compromised the physical structure of cereal, resulting in loose structure arrangement, and the ratio of Fourier transform infrared spectral absorbance at wavelength 1047 cm^−1^ and 1022 cm^−1^, which characterized the short-range order of starch, was significantly reduced (*p* < 0.05). In addition, the proportion of rapidly digestible starch (RDS), the velocity parameter k of digestive kinetics and the predicted glycemic index of cereals were significantly increased by extrusion (*p* < 0.05). Digestibility kinetics showed a total increase of 10.7%, 7.3%, and 5.4% for cereals, along with a sharp rise in digestion rate within the first 15 minutes. The findings revealed that the compromising of starch’s structural integrity and the increase in proportion of RDS not only enhanced overall starch digestibility, but also significantly accelerated its digestion, particularly during the initial 15 min of intestinal digestion.

## Simple Summary

Extruded cereals are widely used in swine production because of their high digestibility and palatability. However, the excessive addition of extrusion cereals can accelerate the release rate of glucose in the digestive tract of pigs, thus breaking the balance of glucose and amino acids during digestion and absorption. Therefore, an in-depth understanding of the effects of extrusion on the dynamics of starch digestion in cereals can provide an important basis for the regulation and optimization of dietary glucose amino acid release patterns. It was found that by destroying the inherent micro-structure of cereals, the extrusion treatment reduced the short-range order of starch, increased the proportion of digestible starch, and significantly improved the digestion rate and predicted glycemic index of starch.

## 1. Introduction

Starch, as the primary constituent of cereals, plays a pivotal role in providing essential energy for both humans and animals. Its overall health maintenance is significantly impacted by its digestion kinetics and position in the gastrointestinal tract. Hence, the digestion rate and extent of enzymes can be used as criteria to categorize starch into rapidly digestible starch (RDS), slowly digestible starch (SDS), and resistant starch (RS) [1]. The extent to which starch digestion occurs can significantly impact the allocation of energy, as well as the metabolism of proteins and lipids [2,3]. According to research findings, glucose released from RDS is quickly absorbed by the proximal small intestine, resulting in an insufficient supply of glucose for the distal portions. Therefore, mucosal cells located in the lower section of the small intestine need to metabolize amino acids to meet their energy demands [4]. Conversely, SDS transports a higher amount of glucose to the distal small intestine, resulting in a reduction of amino acid breakdown within the intestinal mucosa [5]. Additionally, RDS could potentially lead to a rapid glycemic response and large postprandial glycemic fluctuation, whereas SDS is able to maintain a moderate postprandial blood glucose level. RS is not easily digested in the upper digestive system and instead travels to the colon, where it undergoes fermentation by gut bacteria, resulting in the production of short-chain fatty acids (SCFA) that promote a healthy gut [6].

The digestion kinetics of starch can be affected by the structural properties of the food matrix and the starch itself, including the crystalline structure, short-range order, molecular structure (e.g., the size and amount of amylose and amylopectin, the fine structure of amylopectin), morphology, and surface features (e.g., size and shape, the presence of pores and channels) [7]. However, the slow and limited progress of amylolysis is attributed to the dense granular structure and high crystallinity of native starch particles [8]. This hinders the timely release of nutrients from raw starch in both human and animal bodies, leading to a sluggish glycemic response and challenges in maintaining essential body nutrition. Different physical, chemical, and enzymatic methods can be used to modify starch in order to overcome these limitations. Extrusion is a widely applied mechanical process in the food and feed processing sector, aimed at improving the rates of starch digestion.

The findings of various studies have demonstrated that the inclusion of extruded cereals, like extruded corn and extruded broken rice, significantly enhances both nutrient digestibility and feed intake in piglets [9]. However, completely replacing raw corn with extruded corn in the diet of growing pigs increases their metabolic energy expenditure while simultaneously reducing protein deposition by 17.9% and energy utilization efficiency by 1% [3]. This indicates a threshold for glucose intake, beyond which the balance and coordination between nutrient supply and demand were disrupted, leading to suboptimal utilization [10,11]. While the extrusion process enhances starch digestibility and nutrient release rates, excessive inclusion of extruded cereals may disrupt carbon–nitrogen coordination at the nutrient release level, as well as the balance between amino acids and glucose at the transport and absorption levels. Conversely, excessive starch digestion during the gastric and small intestinal phases may impede the delivery of sufficient amounts of soluble carbohydrates to the large intestine, thereby compromising health and hindering optimal energy utilization through alterations in the composition and environment of the large intestinal microbiota. However, studies have demonstrated that the regulation of the glucose release dynamics from diets can enhance growth performance, nitrogen efficiency (5.3%), and protein deposition (8.4%) in pigs [10]. Li et al.‘s research provides further evidence that an optimal release pattern of dietary glucose can optimize the distribution of amino acids in piglets’ portal vein, thereby significantly improving their utilization of dietary protein [12].

Therefore, based on the results of previous study on starch digestion dynamics of different cereals [7], the current study selected corn, wheat, and broken rice commonly used in animal diet with obvious differences in starch digestion dynamics to investigate the impact of extrusion treatment on the physicochemical properties, starch digestion kinetics, and predicted glycemic index of these cereals. Furthermore, the correlation between starch digestion dynamics and physicochemical properties of cereal was analyzed, which provided the basis for the optimization of grain extrusion process and the scientific application of extrusion cereal in livestock and poultry diets.

## 2. Materials and Methods

### 2.1. Materials

The corn was purchased from Yufeng Feed Co. (Acheng, China) and was extruded using a twin-screw extruder (Daze, EXT200, Chengdu, China) with a screw diameter of 197 mm and length of 212 mm. The feed speed and screw speed were set at 2000 kg h^−1^ and 500 rpm, respectively. The extrusion temperature was 140 °C, while the moisture content of the raw material was 15%. The wheat and broken rice were purchased from Henan Shennong Extrude Feed Technology Co., Ltd. (Zhengzhou, China). A twin-screw extruder (Duole, EXP160, Zhengzhou, China) with a screw diameter of 160 mm and length of 1200 mm was used for extrusion; the screw speed was set at 550 rpm. The feed rate of wheat was 1161 kg h^−1^, the extrusion temperature was 130 °C, and the moisture content of the raw material was 15%. The feed rate of broken rice rate was 1338.6 kg h^−1^, the extrusion temperature was 140 °C, and the moisture content of the raw material was 16%. After extrusion, the cereals are air dried at 25 °C for 24 h.

Three samples were randomly collected from extruded and un-extruded cereals and crushed through a 60-mesh sieve for subsequent analysis with three replicates per analysis. In order to eliminate the interference of cereal source on the test results, the cereals before and after the extrusion treatment were the same source and the same batch. At the same time, in order to improve the representativeness of the samples, the newly harvested cereal in 2023, purchased in bulk by typical extrusion enterprises, was selected as the research object.

### 2.2. Scanning Electron Microscopy (SEM)

The cereal samples were affixed onto a circular stub surface using double-sided adhesive carbon tape and then subjected to gold coating in a sputter coater. This allowed for microstructural examination under a scanning electron microscope (Zeiss Gemini SEM500, Oberkochen, Germany) with an acceleration voltage of 2 kV.

### 2.3. Fourier Transform Infrared Spectroscopy (FTIR)

The Fourier transform infrared spectrometer (ALPHA II, Bruker, Germany) was utilized to acquire FT-IR spectra of the cereal pellet samples. The measurements were conducted over a wavelength range from 400 to 4000 cm^−1^. The instrument’s dedicated software, OMNIC 8.2 (Thermo Electron Corporation, Madison, WI, USA), was used to analyze the data. The spectra underwent baseline correction at 1200 and 800 cm^−1^ using a straight line, followed by deconvolution of the spectra. The ratio of the integrated areas of absorption bands at 1047/1022 cm^−1^ was obtained for the deconvoluted spectra [13].

### 2.4. Chemical Composition and Gelatinization Degree (DG)

The AOAC (1990) methods, including 930.15, 942.05, 976.05, and 954.02, were employed to determine the levels of dry matter (DM), ash, crude protein, and crude lipid.

The total starches content was determined by anthrone colourimetric method with the Starch Assay Kit (G0507W, Suzhou Grace Bio-technology Co., Suzhou, China). The amylose content was determined by iodine colourimetric method with the Amylose Assay Kit (G0508W, Suzhou Grace Bio-technology Co., Suzhou, China). Operations were carried out according to kit instructions. The amylopectin is calculated using the following formula:

Amylopectin (%) = (1 − amylose) × 100(1)

The method developed by Birch et al. was employed to assess the DG in cereals [14]. A precise 50 mg sample was weighed and then dissolved in 50 mL of a 0.05 mol/L KOH solution, which was stirred magnetically for 20 min to ensure complete solubilization of the gelatinized starch. After centrifugation at 3400× *g* for 20 min, 1 mL of the supernatant was neutralized with 1 mL of HCl (0.05 mol/L). Subsequently, iodine reagent (0.1 mol/L) and distilled water were added in 1 mL and 8 mL, respectively, followed by measuring the absorbance at a wavelength of 600 nm (A1). In the above steps, KOH (0.05 mol/L) and HCl (0.05 mol/L) were replaced with KOH (0.5 mol/L) and HCL (0.5 mol/L) as sample controls and the corresponding absorbance was recorded as A2. The following formula was used to determine the DG value:

DG = A1/A2 × 100%(2)

where A1 represents the absorbance of the test sample at 600 nm, and A2 denotes the absorbance of the control sample.

### 2.5. The In Vitro Starch Digestion Procedures

The methods described by Englyst et al. [1] were employed to assess the in vitro digestion of cereals, with some minor adjustments. Pepsin and pancreatic enzymes from pigs were used and the incubation temperature was set at 39 °C. In summary, around 1 g of samples, screened through a 1 mm mesh, were incubated in a 10 mL pepsin solution at a pH of 2.0. This solution was composed of 0.05 g of pepsin (P-7000; Sigma Aldrich, Darmstadt, Germany) and 0.05 g of guar gum (P-9000-30-0; Sigma Aldrich, Darmstadt, Germany) dissolved in 0.05 mol/L HCl. The mixture was maintained at 37 °C for 30 min with constant agitation. Then, we added 5 mL of an enzyme mixture containing 0.7 g pancreatin (Sigma P-7545; Sigma Aldrich, Darmstadt, Germany), 0.05 mL amyloglucosidase (A-7095; Sigma Aldrich, Milan, Italy), and 3 mg invertase (Sigma I-4504; Sigma Aldrich, Darmstadt, Germany) to 10 mL of 0.25 mol/L sodium acetate (C_2_H_3_NaO_2_) solution. The pH of the mixture was 6.86. After being incubated for 0, 15, 30, 60, 90, 120, 180, or 240 min, a sample of 0.5 mL was extracted and subsequently treated with absolute ethanol to halt the starch digestion process. Next, the samples were centrifuged at a speed of 3000× *g* for 10 min in order to collect the supernatant. A glucose oxidase kit was used to measure the content of glucose present in the supernatant (Megazyme, Bray, Ireland). The fraction of digested starch for every time interval (DC_t_) was determined using the following formula:

DC_t_ = (amount of released glucose present at time t × 0.9)/TS (3)

where TS represents the total weight of starch in the sample (mg), and 0.9 is the transformation coefficient from starch to glucose (162/180 *w*/*w*).

The average absolute in vitro starch digestion rate, expressed as a fraction of total starch per minute, is calculated by subtracting the proportion of digested starch at each time point from the value at the next time point for each feed.

The formulas provided below were utilized to derive the RDS, SDS, and RS values for different starch fractions:

RDS (%) = (G_20_ − G_0_) × 0.9/TS × 100 (4)

SDS (%) = (G_120_ − G_20_) × 0.9/TS × 100 (5)

RS (%) = 100 − RDS (%) − SDS (%)(6)

where G_0_, G_20_, and G_120_ represent the glucose released (mg) at 0, 20, and 120 min, respectively.

To illustrate the dynamics of in vitro starch digestion, we utilized a first-order exponential model previously established by Goñi et al. [15] for the initial set of data. The form of the first-order equation is:

C_t_ = C_0_ + C_∞_ × (1 − e^−kt^) (7)

where C_t_ represents the quantity of starch digested at a time t, C_0_ represents the quantity digested at the initial time point of 0 min, C_∞_ refers to the maximum digestibility potential of starch, k is the rate at which digestion occurs, and t stands for the duration of the incubation period.

### 2.6. Predicted Glycemic Index (pGI)

The *pGI* for each cereal was determined by calculating the area under the hydrolysis curves (AUC) from 0 to 180 min, following the methodology outlined in a study conducted by Giuberti et al. [16]. Each sample’s hydrolysis index (HI) was calculated by dividing its AUC by the AUC of a corresponding reference product, in this case, white bread. The following formula was used to determine the *pGI*:

*pGI* = 1.013 × HI (8)

### 2.7. Statistical Analysis

Data analysis was conducted using the IBM SPSS 26 software package (IBM Corp., Armonk, NY, USA). The normal distribution and homogeneous variance of all the data were assessed using Levene’s test procedure. Subsequently, one-way analysis of variance (ANOVA) was conducted to analyze the experimental data, followed by Tukey’s post-hoc test. Correlation analysis was used to analyze the relationship between physicochemical properties and digestive properties and predicted glycemic index indices of cereals. Origin 2022 Pro software (OriginLab, Northampton, MA, USA) was used to create a heat map. The mean and standard deviation were used to represent the data. Significance was determined at a *p*-value ≤ 0.05.

## 3. Results

### 3.1. SEM Analysis

**Figure 1:**
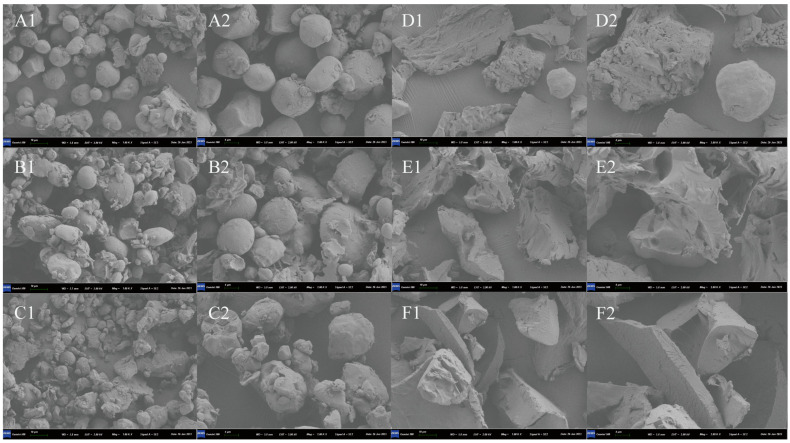
Scanning electron micrograph: (**A**) corn, (**B**) wheat, (**C**) broken rice, (**D**) extruded Corn, (**E**) extruded wheat, and (**F**) extruded broken rice; (1) magnification: 1000× and (2) magnification: 2000×.

The SEM images of various cereals can be observed in Figure 1. Corn particles consist of a mixture of irregularly shaped squares and circles, with some exhibiting rough surfaces and small, round pores (A1, A2). Wheat starch particles are elliptical and nearly circular, and a few are irregular (B1, B2). Broken rice particles exhibit diverse shapes, primarily as irregular polyhedral shapes (C1, C2). The physical integrity of the extruded cereal particles was compromised, exhibiting particle rupture, expansion, structural damage, and substantial degradation. The surface of corn kernels exhibited rough damage, with certain indentations and pores present (D1, D2). Wheat cereals lost their typical elliptical shape and decomposed into a loose, porous irregular structure (E1, E2). Broken rice particles fractured and collapsed into larger flakes or chunks (F1, F2).

### 3.2. FTIR Analysis

**Figure 2:**
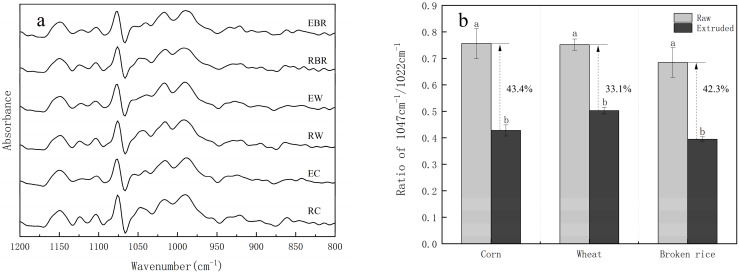
Deconvoluted FT-IR spectrum presented from 800 to 1200 cm^−1^ (**a**) and ratio of 1047 cm^−1^/1022 cm^−1^ (**b**) of raw and extruded samples; error bars show standard error (*n =* 3); bars marked with different letters are significantly different (*p* < 0.05). RC, raw corn; EC, extruded corn; RW, raw wheat; EW, extruded wheat; RBR, raw broken rice; EBR, extruded broken rice.

Figure 2a,b present the deconvoluted spectra of raw cereals and extruded cereals in the region from 1200 to 800 cm^−1^ and ratio of 1047 cm⁻^1^/1022 cm⁻^1^ (R (1047/1022)). The difference in R (1047/1022) between corn, wheat, and broken rice is non-significant. The extrusion processing had significantly reduced the R (1047/1022), with corn and broken rice showing more pronounced effects, with reductions of 43.4% and 42.3%, respectively.

### 3.3. Chemical Composition and DG

The chemical composition and DG analysis results of cereals are presented in Table 1. After extrusion processing, the DM content of the three cereals increased significantly (*p <* 0.05), and the ether extract (EE) content reduced significantly (*p* < 0.05). The CP, ASH, and total starch (TS) contents did not show any significant variation. The amylose content of extruded corn, extruded wheat, and extruded broken rice was significantly increased by 15.8%, 17.1%, and 36.1% (*p* < 0.05), respectively, which resulted in a significant increase in amylose/amylopectin ratio of the three cereals (*p* < 0.05). A significant increase in DG was observed after extrusion treatment for all cereal samples (91.18–95.59%).

### 3.4. In Vitro Starch Digestibility

**Figure 3:**
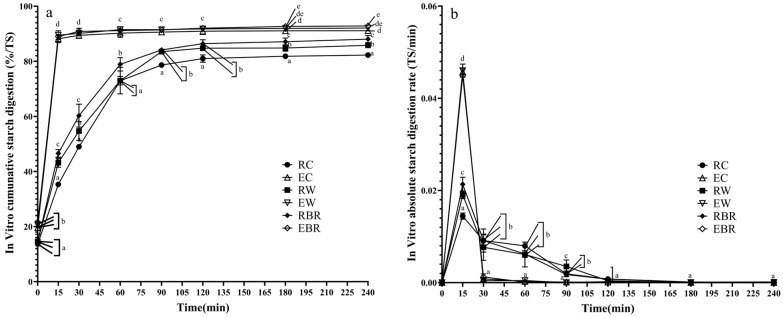
Time course of cumulative in vitro starch digestibility (**a**) and absolute in vitro starch digestion rate (**b**) of each cereal. RC, raw corn; EC, extruded corn; RW, raw wheat; EW, extruded wheat; RBR, raw broken rice; EBR, extruded broken rice. Different lowercase superscripts indicate significant differences (*p* < 0.05).

The time course of the average cumulative (Figure 3a) and absolute curves (Figure 3b) for in vitro starch digestibility showed significant differences among different cereals (*p* < 0.05). The rate of digestion was greatly increased in extruded cereals compared to raw cereals (*p* < 0.05). The digestibility of corn was consistently shown to be lower, while that of wheat and broken rice was demonstrated to be higher among different cereals. The maximum values of in vitro starch digestion curves for all cereals were reached at 15 min of cultivation, with percentages of 89.5%, 89.0%, 88.2%, 46.6%, 43.2%, and 35.3%, respectively. At this time, significant differences were found among the three raw cereals, with broken rice having the highest and corn having the lowest (*p* < 0.05), while no notable variations were found among the different extruded cereals. All of the cereals showed varied degrees of decline in the rate of glucose release after 15 min. Within 15–30 min, the rate of glucose release from the extruded grains dropped sharply, while the decline in the natural grains was relatively moderate. At 30 min of in vitro digestion, the rates of glucose release from the three extruded cereals did not show any significant differences, nor did their cumulative starch digestion rates vary significantly. Glucose release rates among the raw cereals did not differ significantly. However, there were significant differences in cumulative starch digestion rates, with corn showing the lowest digestion rate (49%) and broken rice showing the highest rate (60.3%) (*p* < 0.05). During the in vitro digestion process lasting from 30 to 60 min, the rate of glucose release from extruded cereals tended to become relatively flat, whereas that of raw cereals continued to decrease. From 60 to 120 min during in vitro digestion, the rate of glucose release from extruded cereals was approached to zero, and no significant differences were observed. However, there are still variations in the trends of declining glucose release rates among different raw cereals. The rate of glucose release for all cereals approached zero between 120 and 240 min, with no significant differences.

Table 2 shows the RDS, SDS, and RS fractions of the different cereals. Significant variations were observed in the RDS composition among the three raw cereals, with broken rice having the highest content, followed by wheat, and then, corn (*p* < 0.05). The corn exhibited the highest SDS content (40.18%), while broken rice displayed the lowest SDS content (33.11%) (*p* < 0.05). Compared to broken rice and wheat, corn had a significantly higher RS content (*p* < 0.05). Cereal starch fractions are significantly altered by extrusion processing. Compared with raw cereals, the extrusion of corn, wheat, and broken rice significantly increased the RDS content by 53.2%, 47.6%, and 40.38%, respectively (*p* < 0.05), while the SDS and RS content decreased significantly by 95.6%, 95%, and 92% for SDS, and by 50.1%, 46.2%, and 41.3% for RS, respectively (*p* < 0.05).

After 120 min of digestion, glucose release from all cereals entered a plateau phase, and thus, the kinetic parameters for glucose release were calculated using data from before 120 min. The findings indicated a significant increase (*p* < 0.05) in C_0_ levels for wheat and broken rice when compared to corn. At the same time, no notable variations were observed in the C_∞_ and k values among corn, wheat, and broken rice. After extrusion, there was a significant increase in C_0_ and k values for all three cereals (*p* < 0.05), while C_∞_ remained unchanged.

### 3.5. pGI

Table 2 displays the *pGI* of the cereals. Significant differences were found in the *pGI* among the three raw cereals, with broken rice having the highest content, followed by wheat, and then, corn (*p* < 0.05). After extrusion, the *pGI* of corn, wheat, and broken rice increased significantly by 35.8%, 29.2%, and 22% respectively (*p* < 0.05). The order of the and *pGI* values was as follows: extruded wheat > extruded corn > extruded broken rice.

### 3.6. Relationship Between Chemical Composition, Starch Fractions, and Predicted Glycemic Index

**Figure 4:**
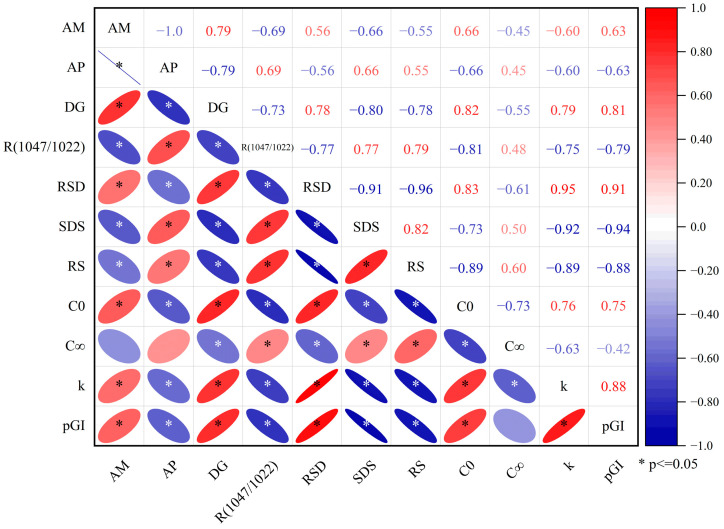
Heat map derived from the Pearson correlation between physicochemical properties, digestive characteristics and predicted glycemic index of cereals. * *p* < 0.05; AM, amylose; AP, amylopectin; DG; degree of gelatinization; R (1047/1022), absorption ratio of 1047 cm⁻^1^/1022 cm⁻^1^; RDS, rapidly digestible starch; SDS, slowly digestible starch; RS, resistant starch; C_0_, the starch digestion ratios at time 0 min; C_∞_, potential digestibility; k, starch digestion rate coefficient; *pGI*, predicted glycemic index.

Figure 4 shows the correlation analysis results between the physicochemical properties, digestive characteristics, and predicted glycemic index of cereals. the *pGI* displayed a significantly positive association with RSD (r = 0.91, *p* < 0.05), k value (r = 0.88, *p* < 0.05), DG (r = 0.81, *p* < 0.05), C_0_ (r = 0.75, *p* < 0.05), and AM (r = 0.63, *p* < 0.05). Conversely, it showed a significant negative relationship with SDS (r = −0.94, *p* < 0.05), RS (r = −0.88, *p* < 0.05), R (1047/1022) (r = −0.79, *p* < 0.05), and AP (r = −0.61, *p* < 0.05). R (1047/1022) was significantly positively correlated with RS (r = 0.79 *p* < 0.05), SDS (r = 0.77, *p* < 0.05), AP (r = 0.69, *p* < 0.05) and significantly negatively correlated with C_0_ (r = −0.81, *p* < 0.05), k (r = −0.75, *p* < 0.05), RSD (r = −0.77, *p* < 0.05), DG (r = −0.73, *p* < 0.05), and AM (r = −0.69, *p* < 0.05). DG was significantly positively correlated with C_0_ (r = 0.82, *p* < 0.05), k (r = 0.79, *p* < 0.05), RDS (r = 0.78, *p* < 0.05), and AM (r = 0.79, *p* < 0.05), and significantly negatively correlated with SDS (r = −0.80, *p* < 0.05), RS (r = −0.78, *p* < 0.05), and AP (r = −0.79, *p* < 0.05).

## 4. Discussion

It is widely acknowledged that the morphology of cereal particles plays a crucial role in determining the digestion pattern of starch. Therefore, SEM was used to examine how the particle composition of various cereals changed both before and after extrusion. It can be seen that the shapes of cereals from different sources are diverse, which is consistent with earlier findings [7]. The protein bodies or broken pieces of the protein matrix that were visible as globular or irregular particles between or attached to the starch granules were caused by the disruption of the protein matrix during milling [17]. Extruded cereal granules were observed to lose physical integrity. According to Zhang et al. [18], native rice starch granules were angular with polyhedral shapes, whereas extruded starch aggregates were continuous and porous. The morphological trait may be the result of protein denaturation, starch gelatinization, and cross-linking of these proteins and starches [19]. Additionally, the cereal experiences high temperature, high shear, and low moisture during extrusion for a brief period. Following this, the pressure is released, and the temperature drops, causing the moisture to flash off. This causes the tightly packed grain to become a spongy, irregular structure [20]. In contrast to the unprocessed cereal, the extruded samples showed a looser structure with big holes that allowed the “endo-corrosion” (from inside out) digestion pattern to pierce deeper into the granules [21]. Since enzymes were easily able to attack this structure, the extruded granules could be digested more rapidly. Meanwhile, the effective surface area can be further increased by cracks and damage in granules. In studies, larger relative surface areas of starch granules increase enzyme contact surfaces [22]. Consequently, this facilitates faster enzyme adsorption and binding rates to macromolecules, thereby accelerating hydrolysis.

Knowing the internal structure of the cereal has a significant influence on the degree of nutrient utilization. In order to learn more about the structure of the extruded starch samples, the FT-IR spectrum was employed in this study. Starch’s sensitive overlap zone for the starch structure was found in the 1200–800 cm^−1^ region of the infrared spectrum. A band in this range is thought to result from stretching vibrations of C-O and C-C [23]. Consequently, this area of absorption was considered as the characteristic fingerprint region for the starch-derived sample, indicating variations in the polymer’s conformation. Deconvolution processing could enhance the resolution of different vibration modes. The variations in absorption at 1047 and 1022 cm^−1^ showed a fair degree of sensitivity to the physical state of starch. The previous literature declared that the crystalline degree and molecule order of starch polymeric substances are related to the peak at 1047 cm^−1^, while the amorphous phase or disordered of starch is indicated by the band intensity at 1022 cm^−1^ [24]. Amorphous layers are commonly regarded as more easily digestible compared to crystalline regions. Hence, the ratio of absorbance at R (1047/1022) has frequently been employed to assess the degree of short-range ordered in starch. A higher R (1047/1022) value indicates a greater extent of crystallization within the starch structure [25]. Raw cereals have a higher absorbance ratio, which suggests that their crystallinity and ordered structure were higher. The differences of R (1047/1022) were not significant for corn, wheat, and broken rice, showing the existence of similarly ordered starch formations. Compared to raw cereals, the peak intensities in extruded cereals show a gradual increase at approximately 1022 cm^−1^, whereas the peak around 1047 cm^−1^ exhibits a more flattens out, which aligns with earlier reports [26]. Three extruded samples had lower R (1047/1022) values than the raw samples. Because of high temperatures and shear forces, the hydrogen bond in starch was likely destroyed, resulting in a disordered structure in the crystal area [27]. Cervantes-Ramirez et al. [28] also reported that the extrusion process destroyed the starch granules in their morphological status, changed the internal crystalline structure, and affected the gelatinization properties.

The findings from the experiment suggest that the chemical makeup of unprocessed grains aligns with the results from the existing literature [7]. After extrusion, the chemical composition and DG of raw cereals are changed. When cereals are extruded, they undergo heat treatment and, subsequently, dry, which is probably why the DM content in the extruded cereals is higher than in the raw cereals [29]. Chien et al. [30] also pointed out that the moisture content of extruded cereals tends to decrease after the extrusion process. This decrease in lipid value may have resulted from the formation of amylose–lipid complexes or from lipids becoming cross-linked with other substances during the extrusion process [31], which reduced their solubility and decreased the apparent lipid content [32]. Another potential explanation could be that the material experiences intense shear forces within the barrel and high pressure at the head of the die, which causes the separation of lipid from the material.

Starch consists of two glucose polymers: amylose and amylopectin. Generally, amylose is typically a linear polysaccharide with 1-4 glycosidic bonds, whereas amylopectin molecules are larger and more highly branched, containing both 1-4 and 1-6 glycosidic bonds. Amylopectin constitutes the predominant portion of raw starch, accounting for 70–80% of the dry weight in wild-type starches, whereas amylose represents a smaller fraction [33]. Since amylose and amylopectin are packed differently, the amylose/amylopectin ratio is a significant indicator of starch and can affect swelling and digestion. According to the results of this experiment, the amylose content of the starch increased in the three raw materials after extrusion treatment, while the amylopectin content decreased, leading to a significant increase in the ratio of amylose to amylopectin. Similar results were also reported by Liu et al. [34], who demonstrated that the amylose content of extruded corn and extruded potato starch increased significantly compared to raw corn and potato starch, with increases of 37.52% and 29.37%, respectively. It occurs because glycosidic bonds are broken during extrusion, causing starch molecules to degrade. Amylose features minimal branching in its long chains, allowing it to experience less interruption compared to amylopectin when subjected to intense shear stress [35]. Moreover, because of the larger molecular size of amylopectin and the higher susceptibility of α-1,6-glycosidic bonds to break compared to α-1,4-glycosidic bonds, numerous branch points readily fracture during extrusion, thereby forming amylose components [36].

Gelatinization refers to the process where raw starch transforms into a cooked, digestible form through the combination of water and heat. After the extrusion treatment, the DG of all cereal samples was significantly increased. When cereals undergo the extrusion process, the interplay of heat, moisture, and mechanical shear rapidly transforms starch granules from a solid state into a molten state [37]. This process involves the disruption of intramolecular and intermolecular hydrogen bonds, resulting in the transformation of starch molecules from their native state to a damaged and gelatinized form. Wang et al. [38] demonstrated that the occurrence of starch gelatinization was accompanied by the disruption of the crystalline structure and degradation of amylopectin double helices, aligning with the findings mentioned above.

Starch is broken down to release glucose, a key nutrient that affects the flow of glucose and the metabolism of insulin in the body. This process plays a significant role in regulating nutrient absorption and overall metabolic functions [39]. Therefore, we conducted in vitro digestion experiments to determine the digestive characteristics of six cereal samples. The findings of the current research revealed that the ranking of raw cereal samples according to their in vitro cumulative starch aligns with the results from earlier investigations [7,16]. Research indicates that the effectiveness of amylases on starch granules is influenced by the internal structure, including amylose and amylopectin content, surface morphology such as granule surface pores, and crystalline type [40]. Compared to wheat and broken rice with a lower amylose content, higher-amylose corn displays lower rates and extents of digestion. Since amylose polymers have a smaller surface area and a higher degree of intramolecular hydrogen bonding than amylopectin, they can form complexes with surfactant compounds [41]. Furthermore, extrusion significantly increases the starch digestibility of cereals. Chien et al. [30] demonstrated that extruded broken rice, corn, and buckwheat had initial hydrolysis rates that were at least 29 times, 6.2 times, and 16.9 times higher than that of native. Similarly, in vivo experiments with pigs showed that extrusion increased the ileal digestibility of corn starch by 0.15 units to 0.98 units [42].The disruption of the dense cereal structure during extrusion results in a spongy matrix, which can be linked to the interplay of shear forces, pressure, and high temperature. Concurrently, starch chains shift from an organized to a disordered configuration, resulting in lower crystallinity [43]. The extrudates exhibit an enhanced surface area and gelatinization level to facilitate swift digestion by amylase. Alternatively, this might have happened due to the reassociation of amylose and amylopectin fragments, leading to the reformation of polymers through weak intermolecular forces during the retrogradation phase of extruded starch [44,45]. Furthermore, the glucose release from extruded cereals reached a plateau after 15 min of hydrolysis, whereas raw cereals require more than 120 min for complete enzymatic digestion. This indicates that the time required for hydrolysis to be finished is significantly reduced by extruded cereals. Kempen et al. [46] noted that the absolute starch release curve over time was more consistent with in vivo portal glucose appearance. The increased peak glucose appearance observed with rapidly digestible starch coincided with an increase in insulin appearing in the portal vein. Overall, extrusion improved the digestibility and digestion rate of starch, which is consistent with the previously proposed microstructure and FTIR results.

Starch can be classified into three categories based on the starch digestion rate: RDS, SDS, and RS. Li and Hu [47] claim that there are parallel and sequential digestion patterns involved in the digestion of RDS and SDS and that these digestion patterns do not begin at the same time. In the early phase of starch digestion, the enzyme directly interacts with the outer layer of starch granules, leading to swift breakdown, whereas only partial contact occurs between the enzyme and the middle and inner regions, resulting in a slower rate of digestion. The findings from our investigation indicated a significant increase in the RDS content of extruded cereals, while the SDS and RS content were significantly reduced. This result aligns with the findings of Lai, et al. [48]. Extrusion caused the starch granules to rupture, making the starch more accessible and allowing starch hydrolysis by exposing interior regions that are more susceptible to enzymatic attack. As a result, previously indigestible SDS and RS are converted into rapidly digestible starch RDS, leading to a significant acceleration in the rate of glucose release. Studies have indicated that the rapid digestion of RDS in the small intestine causes a sharp rise in the concentration of insulin and postprandial blood glucose [16]. The SDS is gradually and thoroughly broken down in the small intestine, whereas RS goes undigested in the upper gastrointestinal tract and is instead fermented by the gut microbiota in the colon, resulting in the production of short-chain fatty acids that contribute positively to colon health [6]. Diets abundant in RS content demonstrate diminished glucose absorption, reduced insulin secretion, and augmented SCFA production [49]. However, diets with high RS contents may lead to a decrease in the digestibility of starch and feed efficiency [50].

In addition, employing a kinetics model for accurately modeling the time-course measurements of starch enzymatic degradation has become common. C_0_ is the starch digestibility of the small intestinal stage at moment 0 and the final digestibility of starch in the gastric stage. The outcomes demonstrated that the C_0_ of the extruded cereals was notably higher than those of the raw cereals, suggesting that the extruded cereals accelerated digestion at the gastric stage. The rate of glucose release during in vitro digestion is indicated by the k-value; the higher the k-value, the more quickly digestion reaches the potential maximum digestibility. The findings indicated that the k values for extruded cereals were considerably greater than those for their raw counterparts. This suggests that the extrusion process notably enhances the rate at which starch is digested in cereals. This could be because extrusion breaks the ordered structure of starch particles, making it easier for enzymes to interact with substrates [22]. Similar findings were reported by AlRabadi et al. [51], indicating that the k value of extrudates made from sorghum starch was greater compared to that of unprocessed starch. Research has indicated that increasing the rate of starch digestion may reduce the quantity of undigested food at the end of the small intestine or proximal colon [51], thereby impacting feed consumption by circumventing the mechanism known as the “ileal brake” [52].

While the enzymatic hydrolysis kinetics suggest that extruded cereal exhibits greater digestibility compared to raw cereal, a study by Chavez-Salazar et al. [53] found that this increased speed of enzymatic hydrolysis leads to an accelerated metabolic rate and elevated postprandial blood glucose response. Hence, we utilized the equation developed by Giuberti et al. [16] to estimate the *pGI* of extruded cereals. As noted by Goñi et al. [15], a *pGI* value above 70 was defined as a high-blood glucose food; values from 55 to 69 indicated a middle-blood glucose food, and those below 55 indicated a low-blood glucose food. After extrusion processing, cereals that were classified as middle-blood sugar food levels were changed to high-blood sugar food levels. After extrusion expansion, RDS accounts for a significant portion of cereal starch (89.17–89.91%) and represents the starch fraction that is rapidly digested in the small intestine. The glucose released is used to supply energy to the upper parts of the small intestine, while the remainder is absorbed into the bloodstream [54], resulting in a rapid increase in glycemic index. Clearly, RS limits the amount of glucose absorbed in the small intestine, leading to a low glycemic index and consequently altering the host’s energy metabolism from glucose to SCFA. Additionally, animal performance and health appear to be strongly correlated with the *pGI* values of feeds. For example, Menoyo et al. [55] found that higher *pGI* cereals improve the insulin response, causing blood glucose to clear quickly and the hunger state to return quickly, which may lead to increased feed intake. Nonetheless, it has been observed that a low *pGI* diet can lead to lower average daily gains, diminished energy, protein retention [2,56], and reduced carcass weights [57] in growing pigs when contrasted with a high *pGI* diet. Furthermore, due to the limited ability of piglets to digest raw starch, it is necessary to use more easily digestible cereal based feeds in pig feeding.

Figure 4 illustrates the heat map and Pearson correlation coefficients for various cereals’ starch fractions, *pGI*, and physicochemical characteristics. A significant negative correlation was observed between DG and the R (1047/1022). This indicated that DG negatively affected the short-range ordered starch structures. This agrees with the literature: Higher shear force extrusion conditions destroyed the starch’s structure, resulting in more amorphous regions and larger weight molecular size distributions [58]. The findings also revealed positive correlations between DG and *pGI*, indicating that the gelatinized component of the cereals had an observable impact on blood glucose levels. The *pGI* showed an inverse relationship with the R (1047/1022), suggesting that the crystalline arrangement of starch considerably hampers its digestibility. Significantly negative correlations were found between the *pGI* and k values and SDS and RS, and significant positive correlations were found with RDS. This result was consistent with the finding that the digestive nutritional starch fractions of RDS increased the rate of enzymatic hydrolysis. This is likely associated with an outcome resulting from the computation of *pGI*, which is inherently linked to measurements of starch fractions. Due to its resistance to enzyme action, several studies also revealed a negative correlation between the amylose content and the starch digestion of starchy materials [7,16]. However, the relationship between starch digestibility and amylose content in this experiment is relatively small compared to the DG, RSD, and k values. This is likely due to the destruction of the crystalline structure of starch in cereal, which significantly reduced the impact of amylose content on starch digestibility.

In order to ensure the accuracy and applicability of the experimental results, all cereals were purchased from larger enterprises in China. The sources of corn and extruded corn, wheat and extruded wheat, and broken rice and extruded broken rice are consistent, which gives them higher representativeness among similar products in the market. The influence of experimental results due to factors such as cereal variety and origin can be effectively avoided by using the same source of cereals for extrusion. However, the experiment may be impacted by the variety, origin, and storage time. Further research and verification are needed to investigate the specific effects of different storage periods among different varieties on experimental results.

## 5. Conclusions

In terms of physical structure, extrusion disrupts the structural integrity of cereal particles, resulting in their collapse into larger flakes with a rough surface and loose texture, increased starch gelatinization, and a reduced short-range ordered structure (R (1047/1022)), thereby facilitating easier digestion. From a chemical composition perspective, extrusion accelerates starch digestion by elevating the proportion of rapidly digestible starch (RDS). The findings demonstrate that extrusion not only enhances starch digestibility but also significantly expedites its digestion rate. Following extrusion, all three cereal starch their maximum digestibility almost 15 min after small intestine digestion, while un-extruded cereals required at least 120 min for intestine digestion. These results provide valuable insights for optimizing grain extrusion technology and the scientific application of extrusion cereal in livestock and poultry feed.

## Figures and Tables

**Table 1:** Chemical composition (% of dry matter), starch fractions (% of total starch) and DG (%) of cereals before and after extrusion treatment.

Items	Corn		Wheat		Broken Rice		*p*-Value
	Raw	Extruded	Raw	Extruded	Raw	Extruded	
DM	89.40 ± 0.12 ^b^	92.79 ± 0.01 ^d^	90.6 ± 0.13 ^c^	93.63 ± 0.08 ^e^	88.17 ± 0.12 ^a^	92.87 ± 0.37 ^d^	<0.001
CP	8.52 ± 0.45 ^bc^	9.04 ± 0.39 ^c^	12.62 ± 0.20 ^d^	12.23 ± 0.47 ^d^	7.57 ± 0.06 ^a^	7.99 ± 0.46 ^ab^	<0.001
EE	2.56 ± 0.03 ^f^	1.66 ± 0.02 ^e^	1.19 ± 0.10 ^d^	0.50 ± 0.09 ^b^	0.94 ± 0.12 ^c^	0.21 ± 0.03 ^a^	<0.001
Ash	1.78 ± 0.30 ^b^	2.17 ± 0.42 ^b^	1.79 ± 0.05 ^b^	1.87 ± 0.03 ^b^	0.37 ± 0.02 ^a^	0.35 ± 0.02 ^a^	<0.001
TS	65.63 ± 2.83 ^b^	64.56 ± 1.73 ^ab^	61.44 ± 2.31 ^ab^	60.21 ± 3.85 ^a^	74.00 ± 3.01 ^c^	71.20 ± 2.74 ^c^	<0.001
AM	33.69 ± 1.18 ^b^	40.03 ± 0.79 ^c^	29.75 ± 1.51 ^a^	35.90 ± 1.38 ^b^	22.88 ± 1.50 ^a^	35.82 ± 0.71 ^b^	<0.001
AP	66.31 ± 1.18 ^b^	59.97 ± 0.79 ^a^	70.25 ± 1.51 ^c^	64.10 ± 1.38 ^b^	77.12 ± 1.50 ^d^	64.18 ± 0.71 ^b^	<0.001
AM/AP	0.51 ± 0.03 ^c^	0.67 ± 0.02 ^e^	0.42 ± 0.03 ^b^	0.56 ± 0.32 ^d^	0.30 ± 0.03 ^a^	0.56 ± 0.12 ^d^	<0.001
DG	23.25 ± 1.51 ^a^	95.59 ± 0.41 ^d^	38.10 ± 1.57 ^b^	91.18 ± 0.79 ^c^	24.75 ± 0.66 ^a^	91.28 ± 2.01 ^c^	<0.001

Mean of three replicates. Significant differences (*p* < 0.05) are indicated by values in each column that are followed by different letters. (*p* < 0.05). Abbreviations: DM, dry matter; CP, crude protein; EE, ether extract; TS, total starch; AM, amylose; AP, amylopectin; AM/AP, ratio of amylose to amylopectin content; and DG, degree of gelatinization.

**Table 2:** Proportion of starch fractions, digestive kinetic parameters, and pGI in cereals before and after extrusion.

Items	Corn		Wheat		Broken Rice		*p*-Value
	Raw	Extruded	Raw	Extruded	Raw	Extruded	
RDS	41.67 ± 0.78 ^a^	89.17 ± 0.96 ^d^	47.07 ± 1.19 ^b^	89.91 ± 0.76 ^d^	53.30 ± 1.11 ^c^	89.40 ± 0.43 ^d^	<0.001
SDS	40.18 ± 1.20 ^d^	1.76 ± 0.31 ^a^	37.71 ± 0.87 ^c^	1.88 ± 0.64 ^a^	33.11 ± 167 ^b^	2.62 ± 0.26 ^a^	<0.001
RS	18.16 ± 1.21 ^c^	9.06 ± 1.19 ^a^	15.3 ± 0.35 ^b^	8.22 ± 0.26 ^a^	13.59 ± 1.40 ^b^	7.98 ± 0.60 ^a^	<0.001
C_0(120 min)_	12.98 ± 0.20 ^a^	19.58 ± 2.34 ^c^	15.52 ± 0.95 ^b^	20.50 ± 0.62 ^c^	15.07 ± 0.89 ^b^	21.58 ± 0.71 ^c^	<0.001
C_∞(120 min)_	73.29 ± 1.28 ^b^	70.75 ± 1.50 ^ab^	72.13 ± 2.47 ^ab^	70.76 ± 1.19 ^ab^	72.39 ± 1.01 ^ab^	69.80 ± 0.52 ^a^	0.100
k_(120 min)_	0.02 ± 0.00 ^a^	0.23 ± 0.02 ^b^	0.03 ± 0.00 ^a^	0.26 ± 0.05 ^b^	0.03 ± 0.00 ^a^	0.22 ± 0.11 ^b^	<0.001
*pGI*	53.07 ± 0.61 ^a^	82.68 ± 1.25 ^d^	58.64 ± 0.71 ^b^	82.85 ± 2.00 ^d^	63.13 ± 2.74 ^c^	81.00 ± 1.09 ^d^	<0.001

Mean of three replicates. Significant differences (*p* < 0.05) are indicated by values in each column that are followed by different letters. (*p* < 0.05). Abbreviations: RDS, rapidly digestible starch; SDS, slowly digestible starch; RS, resistant starch; C_0_, the starch digestion ratios at time 0 min; C∞, potential digestibility; k, starch digestion rate coefficient.; *pGI*, predicted glycemic index.

## Data Availability

Data are contained within the article.
